# The Controversial Link between Hepatitis B Virus and Celiac Disease

**Published:** 2010-12-01

**Authors:** Aurora Burgos, Pedro Emilio Bermejo

**Affiliations:** 1 Hospital Universitario La Paz, Servicio de Aparato Digestivo, Madrid, Spain; 2 Hospital Universitario Puerta de Hierro, Servicio de Neurología, Madrid, Spain

Dear Editor,

Celiac disease (CD) is an autoimmune disease resulting in inflammatory destruction of small intestinal mucosa after the ingestion of gluten in genetically susceptible individuals. The study of Leonardi and La Rosa [1] tries to establish a possible link between hepatitis B virus (HBV) infection and celiac disease. This is really interesting because only few cases have been described so far in the literature. However, there are two controversial points to be discussed in more detail-the development of CD after HBV infection or after treatment of chronic hepatitis with interferon, and an inadequate response to hepatitis B immunization in patients with CD.

In the first case, several triggers developing an immunologic intolerance to gluten in susceptible patients have already been described including HBV infection, natural interferon released in response to infection or exogenous interferon used for treatment of viral hepatitis [2][3]. However, we cannot establish definitive conclusions about the relationship between these two entities-HBV and CD. For this reason, in this study [1], the authors think that it is not mandatory to check for specific CD antibodies before beginning the treatment or during the follow-up; but this is still a point for discussion and other studies have proposed that these antibodies should be checked [3].

In the second case, data regarding the response to HBV vaccine in CD suggest that non-response to vaccine is higher in CD patients than in control subjects (67.5% vs 85.2%) [4]. The majority of these studies included adult celiac patients; it has been shown that younger subjects have a better response. Apart from the age, this poor response has been particularly associated with the major histocompatibility complex (HLA) DQ2, DR3 and DR7 which are closely associated with CD as well. Another study [5], demonstrated a correlation between the disease activity (by measuring serum titers of anti-transglutaminase) and the development of an antibody response to HBV vaccine. A gluten-free diet might have a primary role and these authors suggested that HLA DQ2 per se, was not a good indicator for poor response to HBV vaccine.

In conclusion, we think that checking for specific CD antibodies should be recommended before beginning of the treatment with interferon or during the follow-up, although it is not mandatory since it is still a controversial issue. Finally, gluten-free diet may ameliorate the immune response to HBV vaccine in celiac patients; assessment of this response should be routinely considered in children newly diagnosed with CD.
